# MicroRNA-146a Represses Mycobacteria-Induced Inflammatory Response and Facilitates Bacterial Replication via Targeting IRAK-1 and TRAF-6

**DOI:** 10.1371/journal.pone.0081438

**Published:** 2013-12-16

**Authors:** Shuo Li, Yan Yue, Wei Xu, Sidong Xiong

**Affiliations:** 1 Institute for Immunobiology and Department of Immunology, Shanghai Medical College, Fudan University, Shanghai, People's Republic of China; 2 Jiangsu Provincial Key Laboratory of Infection and Immunity, Institutes of Biology and Medical Sciences, Soochow University, Suzhou, People's Republic of China; University of California Merced, United States of America

## Abstract

**Background:**

Apart from triggering host immune responses, macrophages also act as a major reservoir for mycobacteria. For better survival, mycobacteria have evolved various mechanisms to modulate the production of proinflammatory cytokines in macrophages, and manipulation of micro-RNA (miRNA) expression has been considered as an important one.

**Methodology/Principal Findings:**

In this study, we found that miR-146a expression was significantly increased in a time- and dose-dependent manner in mycobacteria-infected macrophages. It could obviously reduce the induction of proinflammatory cytokines TNF-α, IL-1β, IL-6 and chemokine MCP-1 by targeting interleukin-1 receptor-associated kinase-1 (IRAK-1) and TNF receptor-associated factor-6 (TRAF-6), two key elements involved in the TLR/NF-κB signaling pathway cascades. Consistent with the anti-inflammation effect, a higher bacterial burden was seen in miR-146a mimics-treated macrophages.

**Conclusion/Significance:**

Here, we demonstrated that mycobacteria-induced miR-146a could modulate inflammatory response by targeting IRAK1 and TRAF6 and facilitate mycobacteria replication in macrophages.

## Introduction

Tuberculosis (TB), a highly infectious respiratory disease caused by *Mycobacterium tuberculosis* (*Mtb*) infection, has become a major threat to public health. Despite of various anti-mycobacterial therapies, TB remains one of the world's major causes of illness and death. Approximately one third of the world's population is thought to be infected with *Mtb*, and more than 9 million develop “active” TB each year [1], [2]. As the first line of host defense, macrophages are responsible for intracellular killing of *Mtb*, paradoxically, they are also the principal target cells [3]–[5]. Thus, a better understanding of the interaction between *Mtb* and macrophages may contribute to the better control of TB.

It has been well-accepted that *Mtb* has evolved a serious of strategies to subtly modulate host immunity and create a microenvironment favoring its replication and growth. Of which, regulation of microRNAs (miRNAs) expression has been considered as an important one. MiRNAs are non-coding, single-stranded RNAs of ∼22 nt in length that regulate gene expression by triggering mRNAs degradation or inhibiting translation [6], [7]. Recently, accumulating evidence has demonstrated the miRNAs possess immune regulation ability in infectious and autoimmune diseases [8], [9]. Of which, miR-146a has been reported to regulate innate immune responses [10], and its up-regulation is associated with tissue chronic inflammation [11]. Liu and his colleagues [12] have reported that miR-146a could be induced by Helicobacter pylori infection and negatively modulate host proinflammatory cytokine production. Rom and his colleagues [13] have shown that up-regulated miR-146a participated in the HIV-mediated chronic inflammation of brain by targeting chemokine MCP-2. In accordance, our previous data showed that miR-146 expression significantly increased in mouse macrophages post *Mycobacterium bovis* Bacille Calmette-Guérin (BCG) infection (unpublished data), suggesting that miR-146a may play a role in TB-associated inflammation.

In this study, we investigated the dynamic expression of miR-146a in mycobacteria-infected macrophages and found that miR-146a was robustly up-regulated in a time- and bacterial dose-dependent manner. We have also found that miR-146a could significantly suppress the induction of proinflammatory cytokines TNF-α, IL-1β, IL-6 and chemokine MCP-1 and lead to a higher bacterial burden in infected macrophages. This anti-inflammation effect of miR-146a might be mainly mediated by its targeting of IRAK-1 and TRAF6, two key elements in NF-κB proinflammatory signaling pathway. In conclusion, this study provided clues to the regulation role of miR-146a in mycobacteria-triggered inflammation, and gave a hint that modulating miR-146a expression may represent a new therapeutic approach against TB.

## Results

### MiR-146a was up-regulated in mycobacteria-infected macrophages

**Figure 1:**
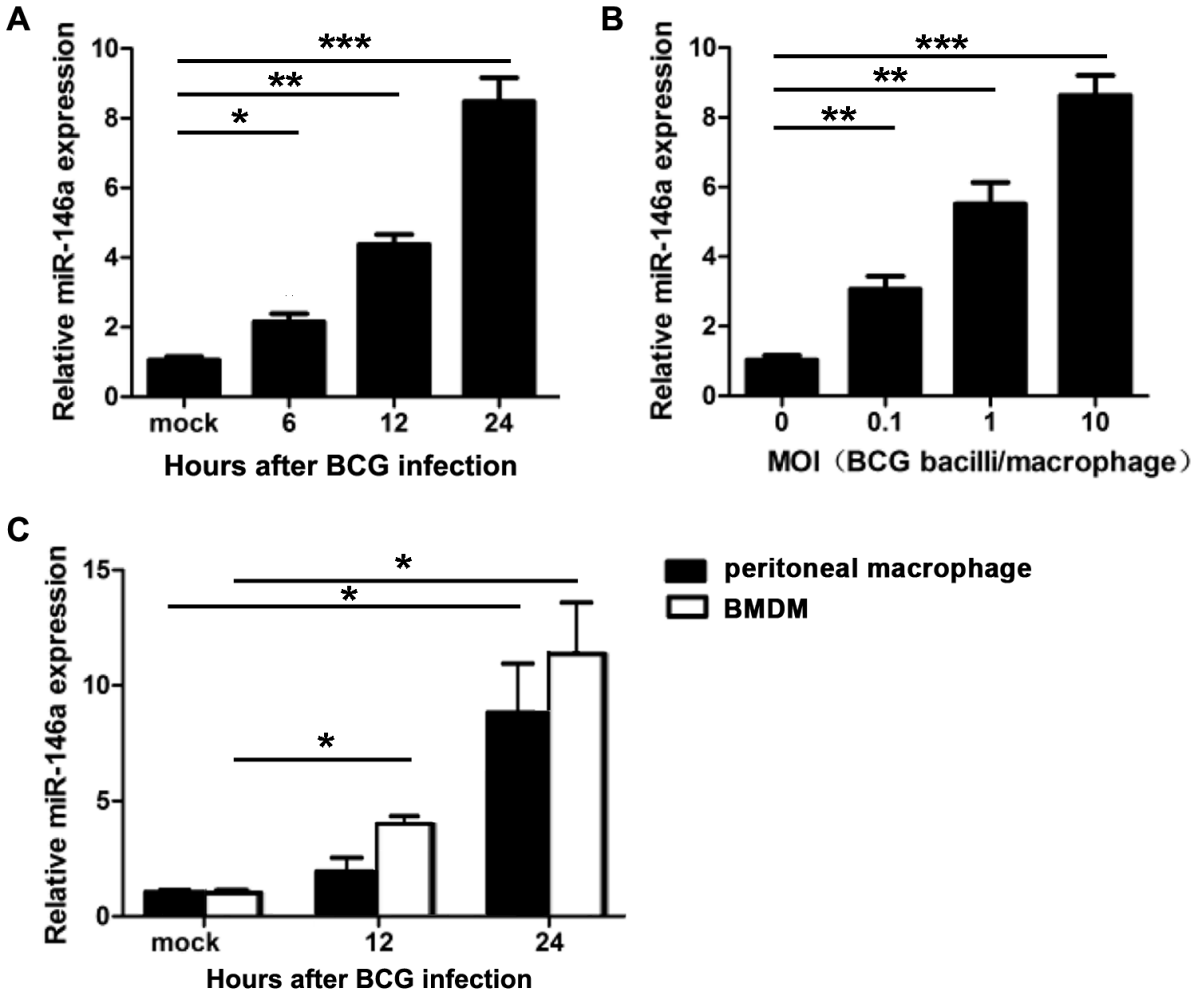
Up-regulation of miR-146a in mycobacteria–infected macrophages. (A) RAW264.7 cells were infected with mycobacteria at a MOI of 10 for indicated time points. The level of miR-146a was measured by real-time PCR. (B) RAW264.7 cells were infected with mycobacteria at the indicated MOI for 24 h, the level of miR-146a was measured by real-time PCR. (C) Mouse peritoneal macrophages and BMDMs were infected with mycobacteria at a MOI of 10 for the indicated time points, and the level of miR-146a was measured by real-time PCR. Data were means ± SD of three independent experiments. *P<0.01, **P<0.01,***P<0.001.

We firstly detected the expression kinetics of miR-146a in mycobacteria-infected macrophages by real-time PCR. As shown in Fig. 1A, up-regulated miR-146a was observed as early as 6 h post-infection, and then gradually increased and achieved maximum at 24 h, about 8.3 fold higher that of 0 h. Furthermore, miR-146a expression was induced by mycobacteria infection at a dose of MOI 0.1 and correspondingly augmented when the dose increased. In accordance, increased miR-146a was also evidenced in mycobacteria-infected primary peritoneal macrophages and bone marrow-derived macrophages (Fig. 1C). These data indicated that miR-146a could be induced in mycobacteria-infected macrophages in a time- and dose-dependent manner.

### MiR-146a suppressed the inflammatory response in mycobacteria-infected macrophages

**Figure 2:**
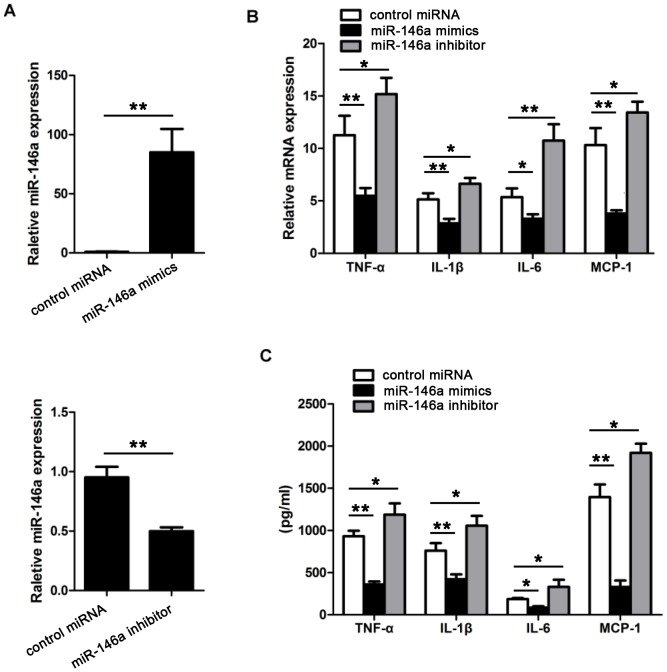
The impact of miR-146a on mycobacteria-triggered production of proinflammatory cytokines and chemokine. (A) Level of miR-146a in miR-146a mimics- or inhibitor-treated macrophages. (B) MRNA expression of proinflammatory cytokines TNF-α, IL-1β, IL-6 and chemokine MCP-1 in miR-146a mimics- or inhibitor-treated macrophages post mycobacteria infection. (C) Protein expression of proinflammatory cytokines TNF-α, IL-1β, IL-6 and chemokine MCP-1 in miR-146a mimics- or inhibitor-treated macrophages post mycobacteria infection. Data were means ± SD of three independent experiments. *P<0.05, **P<0.01.

To investigate the impact of miR-146a on mycobacteria-triggered inflammation, miR-146a inhibitor or mimics was applied and induction of proinflammatory cytokines TNF-α, IL-1β, IL-6 and chemokine MCP-1 in mycobacteria-infected macrophages was detected by real-time PCR and ELISA assays. As shown in Fig. 2, compared with control group, miR-146a inhibition dramatically increased mycobacteria-induced production of proinflammatory cytokine and chemokine, the up-regulation rates ranged from 20% to 100%. In contrast, miR-146a mimics significantly decreased their expression. These data indicated that miR-146a negatively regulated the mycobacteria-induced inflammatory response in macrophages.

### MiR-146a facilitated mycobacterial replication in macrophages

**Figure 3:**
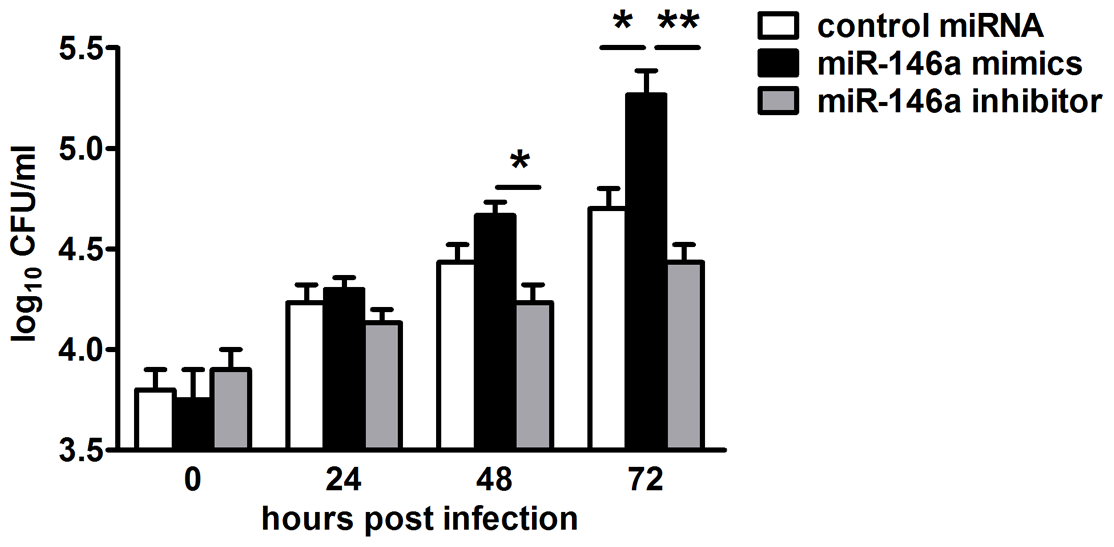
Effect of miR-146a on bacterial burden in mycobacteria-infected macrophages. RAW264.7 cells were transfected with control miRNA, miR-146a mimics or miR-146a inhibitor for 24 h, and then infected with mycobacteria at a MOI of 10 for 6 h. Bacterial burden was detected by CFU assays. Data were representative of three independent experiments. *P<0.05, **P<0.01.

Since proinflammatory cytokines and chemokines are important for mycobacteria clearance, we next investigated whether miR-146a could affect the bacterial burden in infected macrophages. RAW264.7 cells transfected with miR-146a inhibitor, mimics or control miRNA were infected with mycobacteria for 6 h and the kinetics of bacterial burden were monitored for 72 h. As shown in Fig. 3, compared with miR-146a inhibitor-treated group, mycobacterial burden in miR-146a mimics-treated group obviously increased as early as 48 h post infection, and further augmented as time goes to 72 h. Compared with control group, a significantly raised bacteria growth was also shown in the mimics-treated group. These data suggested that miR-146a would facilitate the mycobacterial replication in macrophages.

### MiR-146a targeted IRAK-1 and TRAF-6 in mycobacteria-infected macrophages

**Figure 4:**
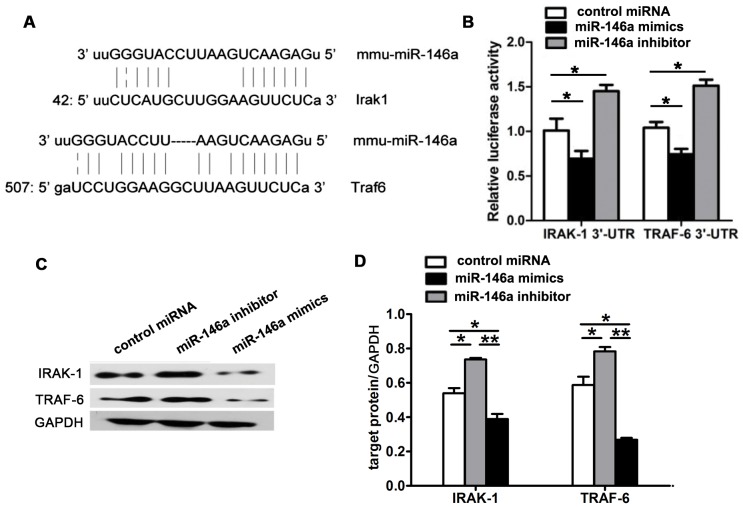
miR-146a targeted IRAK-1 and TRAF-6 in mycobacteria-infected macrophages. (A) Sequence alignment of miR-146a and its target sites in 3′-UTR segments of IRAK-1 and TRAF-6. (B) RAW264.7 cells were co-transfected with miR-146a mimics, inhibitor or control miRNA and the reporter plasmid containing 3′-UTR segment of IRAK-1 or TRAF-6, as well as an endogenous control Renilla luciferase plasmid pRL-TK for 48 h, and then the relative luciferase activity in each group was analyzed. (C) RAW264.7 cells were transfected with miR-146a mimics, inhibitor or control miRNA for 24 h, and then infected with mycobacteria for 6 h. The expression of IRAK-1, TRAF-6 and GAPDH was detected by western blot. (D) Statistical analysis of ratio of IRAK-1 or TRAF-6 to GAPDH band intensity in indicated siRNA-treated RAW264.7 cells post mycobacteria infection. *P<0.05,**P<0.01.

To further explore the potential molecular mechanism underlying the anti-inflammation effect of miR-146a, the target genes of miR-146a were predicted using the programs TargetScan and miRanda. Among them, IRAK-1 and TRAF-6, two molecules involved in pro-inflammatory NF-κB signaling pathway, attracted our more attention (Fig. 4A). To confirm that IRAK-1 and TRAF-6 were regulated by miR-146a, their 3′-UTR segments were respectively cloned into a reporter plasmid downstream from luciferase, and reporter assays were then performed. It was found that miR-146a inhibitor caused substantial up-regulation of IRAK-1 and TRAF-6 reporter gene expression (Fig. 4B), the luciferase activities enhanced from about 1.0 to 1.5 (IRAK-1) and 1.6 (TRAF-6). Conversely, miR-146a mimics robustly reduced IRAK-1 and TRAF-6 reporter gene expression, the luciferase activities were only 0.6 (IRAK-1) and 0.7 (TRAF-6), significantly lower than those in control and mimics-treated groups. These data suggested that miR-146a suppressed IRAK-1 and TRAF-6 by direct binding to their 3′-UTR segments. Furthermore, miR-146a inhibitor led to an obvious up-regulation of IRAK1 and TRAF6 proteins, as shown by western blot results (Fig. 4C), the band intensities were 0.74 and 0.77 respectively, significantly higher than those in control group (0.53 and 0.58). While in miR-146a mimics treated group, the densities of western blot bands significantly declined to 0.38 and 0.26 (Fig. 4D).

**Figure 5:**
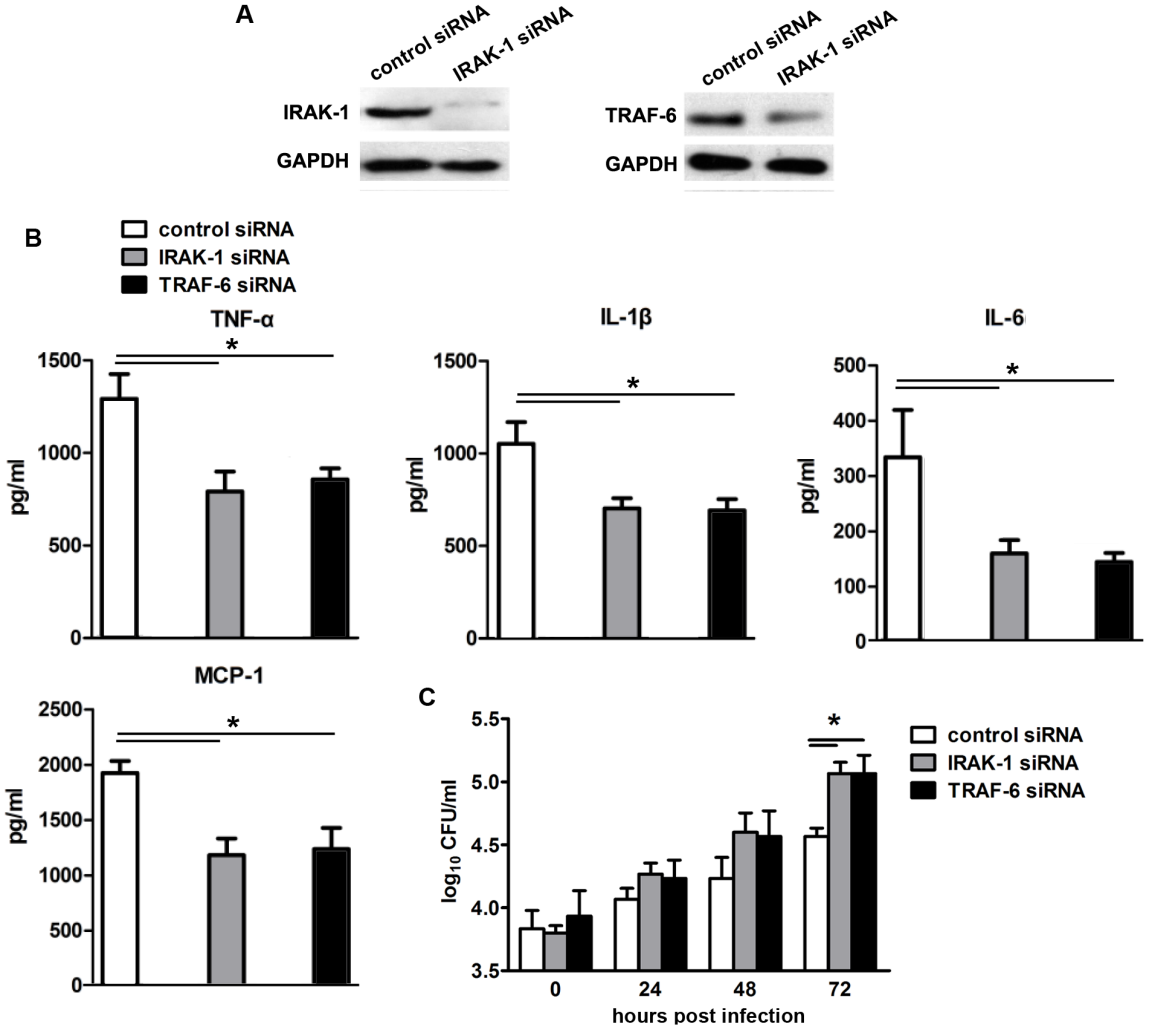
IRAK-1 and TRAF-6 were the functional targets of miR-146a in mycobacteria-infected macrophages. (A) RAW264.7 cells were transfected with IRAK-1- or TRAF-6-specific siRNA, and then the expression of IRAK-1 and TRAF-6 was determined by western blot. (B) RAW264.7 cells were co-transfected with miR-146a inhibitor and indicated siRNAs. Following mycobacteria infection, the level of TNF-α, IL-1β, IL-6 and MCP-1 in the supernatant was measured by ELISA assays. (C) Bacterial burden in RAW264.7 co-transfected with miR-146a inhibitor and indicated siRNAs during mycobacteria infection. *P<0.05.

To confirm that IRAK-1 and TRAF-6 were the functional targets of miR-146a, we knocked down IRAK-1 or TRAF-6 expression by their respective specific siRNAs, and then evaluate miR-146a effect on the proinflammatory cytokine production and bacterial burden in infected macrophages. As shown in Fig. 5, knock down of IRAK-1 or TRAF-6 resulted in the drastically decrease of the proinflammatory cytokines TNF-α, IL-1β, IL-6 and chemokine MCP-1 in miR-146a-inhibited macrophages, accompanied with the increased bacterial burden. These data indicated that miR-146a could reduce the proinflammatory cytokines and facilitate mycobacterial replication by functionally targeting IRAK-1 and TRAF-6.

## Discussion

It has been well-acknowledged that macrophages play a dual role in the pathogenesis of TB. They have been implicated as the predominant protective innate immune cells by phagocytosing, killing mycobacteria and initiating the inflammatory responses. However, they are also the primary target cells of mycobacteria and provide sanctuary during the dormancy period. Interestingly, mycobacteria have evolved a series of modulating mechanisms to ensure their persistence within the cells, like inhibiting phagolysosome fusion and neutralizing the acidic environment of the phagolysosomal compartment. Furthermore, mycobacteria are also endowed with the unique capacity to modulate inflammation, such as regulation of the immune cell recruitment [14] and production of anti-inflammatory cytokines including IL-10 [15] and TGF-β [16].

Recently, miRNAs have been indicated to be one important mechanism utilized by mycobacteria to skew the immune microenvironment to their benefit. It has been reported that inductive miR-21 impaired host anti-mycobacterial T cell responses by targeting IL-12 and Bcl-2 [17]. MiR-29 has been shown to suppress host immune responses by targeting IFN-γ [18]. Therefore, identification and target of immune evasion-associated miRNAs may help to better control mycobacterial replication and represent a novel therapeutic strategy against TB.

Although multiple miRNAs are induced in innate immune cells, miR-146a has been consistently observed in many experimental settings [19]–[21]. Since its first report in LPS-stimulated human promonocyte THP-1 cells [22], accumulating evidence has been shown that miR-146a could be induced by other inflammatory TLR agonists [2], viruses [23] as well as bacteria [24]. Consistent with previous studies [25], here we found that miR-146a was up-regulated in mycobacteria-infected macrophages and appeared in a time- and dose-dependent manner. This increased expression might be ascribed to the activation of TLRs and NF-κB signaling pathway by bacteria components, as has since been shown in studies [26], [27].

Mycobacterium has been demonstrated to induce proinflammatory gene transcription in macrophages through the TLR2 and TLR4, which initiate signaling cascades and lead to NF-κB activation [28]. Recent reports have shown that in addition to the regulation of TLR on mRNA expression [29], [30], miRNAs could conversely modulate the activation of TLR signaling pathways and influence the resultant immune responses [31]. In this study, we found that miR-146a targeted IRAK-1 and TRAF-6, two central adaptor kinases in the downstream signaling cascade of TLR, and led to obvious reduction of proinflammatory cytokines TNF-α, IL-6, IL-1β and chemokine MCP-1. Our results agreed with those of other studies, in which IRAK-1 and TRAF-6 were also shown to be the targets of miR-146a, and their down-regulation robustly reduced the inflammatory responses [32], [33].

Based on these data, a subtle negative feedback regulatory loop of TLR and miR-146a could be speculated in mycobacteria-infected macrophages. Mycobacterial components firstly activate TLR signaling pathways and cause the miR-146a expression via NF-κB pathway. The inductive miR-146a in turn modulates TLR signaling pathway by targeting its downstream molecules of IRAK-1 and TRAF-6, and then limits inflammation. This, on the one hand, could avoid the additional damage caused by excessive immune responses; on the other hand it could also be utilized by mycobacteria for better replication. Actually, we did observed a slightly, but not significantly, decreased mycobacterial burden in miR-146a inhibitor-treated macrophages compared with control group. We attributed this no significant difference to the complexity of mycobacteria control. In fact, besides of the proinflammatory cytokines, other macrophage-derived factors such as Nitric oxide [34], cytokines such as TGF-β [35] as well as cholesterol oxidase [36] are also involved in limiting mycobacterial replication. Thus, miR-146a inhibition alone may not sufficiently cause an obvious decrease in the bacterial burden. Fortunately, a significantly increased burden was evidenced in miR-146a mimics-treated group. This might be explained by the fact that the over-expressed miR-146a amplified and highlighted the facilitation effect of miR-146a on mycobacterial replication. Similar results were also observed by Liu et al [12].

In conclusion, our results demonstrated that mycobacteria infection could induce the miR-146a expression in macrophages, and miR-146a might function as a novel negative regulator of mycobacteria-triggered inflammatory response via targeting IRAK-1 and TRAF-6. This study may present clues for understanding the immune-regulation role of miR-146a in mycobacteria infection, and may also provide a potential therapeutic target for TB.

## Materials and Methods

### Ethics statement

This study was carried out in strict accordance with the recommendations in the Guide for the Care and Use of Medical Laboratory Animals (Ministry of Health, P. R. China, 1998). The protocol was approved by Shanghai Medical Laboratory Animal Care and Use Committee (Permit number: SYXK 2010-0049) as well as the Ethical Committee of Fudan University (Permit number: 2010031).

### Mice and mycobacterium

C57BL/6 mice (6 to 8 week-old) were purchased from Experimental Animal Centre of Chinese Academy of Sciences (Shanghai, P. R. China) and bred in the specific pathogen-free facility. BCG (Denmark strain 1331) was grown in Middlebrook 7H9 broth (Difco, BD Biosciences) supplemented with 10% Middlebrook OADC enrichment (Difco, BD Biosciences), 0.002% glycerol, and 0.05% Tween 80 at 37°C until the mid-exponential phase and then stored in −70°C.

### Cell culture and transfection

Mouse primary peritoneal macrophages and bone marrow-derived macrophages (BMDMs) were isolated according to the previous reports [37], [38]. The murine macrophage cell line RAW264.7 (ATCC TIB-71) was cultured in RPMI 1640 medium supplemented with 10% fetal calf serum (FBS), 10 mM L-glutamine, 100 U/ml penicillin, 0.1 mg/ml streptomycin (Invitrogen) in a humidified incubator at 37°C and 5% CO_2_.

The murine miR-146a inhibitor, mimics and negative control were obtained from Ribobio (Guangzhou, China). The siRNAs targeting IRAK-1 or TRAF-6 were obtained from Santa Cruz. Above miRNAs or siRNAs were transfected to RAW264.7 cells respectively for 48 h using Amaxa Cell Line Nucleofector Kit V (Lonza, Switzerland) according to the manufacturer's protocol.

### Enzyme-linked immunosorbent assay

To assess the level of TNF-a, IL-1β, IL-6 and MCP-1 in the culture supernatant, ELISA assays were performed with relative ELISA Kits (eBioscience, USA) according to the manufacturer's instructions.

### Mycobacteria infection and CFU determination

In most experiments, RAW264.7 cells (2×10^5^) were infected with mycobacteria at a MOI of 10 for 6 h. To detect the kinetic expression of miR-146a, cells were infected with mycobacteria at MOI 10 for different periods (0, 6, 12 or 24 h) or infected with different doses of mycobacteria (MOI 0, 0.1, 1, 10) for 24 h.

To determine the intracellular bacterial growth, RAW264.7 cells were lysed at 0, 24, 48 and 72 h post mycobacteria infection. And then the lysate was serially diluted and plated on kanamycin-supplemented 7H11 agar plates. Colony forming units (CFU) were evaluated by counting individual colonies after 3 week of growth at 37°C.

### Quantitative real-time PCR

The total RNA was extracted with TRIzol reagent (Invitrogen) following the manufacturer's instructions. To detect the expression of miR-146a, total RNA was reversely transcribed with miR146a-specific stem-loop RT primer into cDNA, and then subjected to SYBR green real-time PCR with specific primers for miR-146a and house-keeping gene U6 obtained from Ribobio (China). To detect the expression of proinflammatory cytokines and chemokine, total RNA was reversely transcribed to cDNA with oligo dT primer, and then subjected to SYBR green real-time PCR. Real-time primers were designed and sequences were shown in Table 1. Gene expression was analyzed using the 2^−ΔCtΔCt^ method.

**Table 1:** Real-time primers sequences.

		Primer sequence (5′-3′)
TNF-α	sense	5′-CCTGTAGCCCACGTCGTAG-3′
	anti-sense	5′-GGGAGTAGACAAGGTACAACCC-3′
IL-1β	sense	5′-GAAATGCCACCTTTTGACAGTG-3′
	anti-sense	5′-CTGGATGCTCTCATCAGGACA-3′
IL-6	sense	5′-TAGTCCTTCCTACCCCAATTTCC-3′
	anti-sense	5′-TTGGTCCTTAGCCACTCCTTC-3′
MCP-1	sense	5′-TTAAAAACCTGGATCGGAACCAA-3′
	anti-sense	5′-GCATTAGCTTCAGATTTACGGGT-3′
GAPDH	sense	5′-CTGCACCACCAACTGCTTAG-3′
	anti-sense	5′-GTCTGGGATGGAAATTGTGA-3′

### Luciferase assay

For luciferase reporter experiments, the 3′-UTR segments of IRAK-1 and TRAF-6 predicted to interact with miR-146a were amplified by PCR and inserted into pGL3 vector immediately downstream from the stop codon of luciferase (Promega). RAW264.7 cells were cotransfected in 12-well plates with 0.4 µg of the firefly luciferase report vector and 0.08 µg of the control vector containing *Renilla* luciferase, pRL-TK (Promega), as well as with 100 nM miR-146a mimics, inhibitor or control miRNA. 48 h later, luminescence was detected using the Dual-Luciferase Reporter Assay System (Promega, USA) according to the protocol. Data were normalized to the Renilla luminescence and presented relative to control miRNA transfected group.

### Western blot analysis

The expression of IRAK-1 and TRAF-6 in miR-146a mimics, inhibitor or control miRNA-treated macrophages following mycobacteria infection was detected by western blot as described previously [12].

### Statistical analysis

The experimental data in this study were presented as the means ± SD of three independent experiments. The statistical significance of the differences in the experimental data was valued by ANOVA followed by Tukey's post hoc test. The statistical significance level was set as *P<0.05; **P<0.01, ***P<0.001.
